# Supplementary figures and images for: Genome-wide quantification of contributions to sexual fitness identifies genes required for spore viability and health in fission yeast
Source: PLoS Genet. 2022 Oct 27;18(10):e1010462. doi: 10.1371/journal.pgen.1010462 (PMC9668190; doi:10.1371/journal.pgen.1010462)

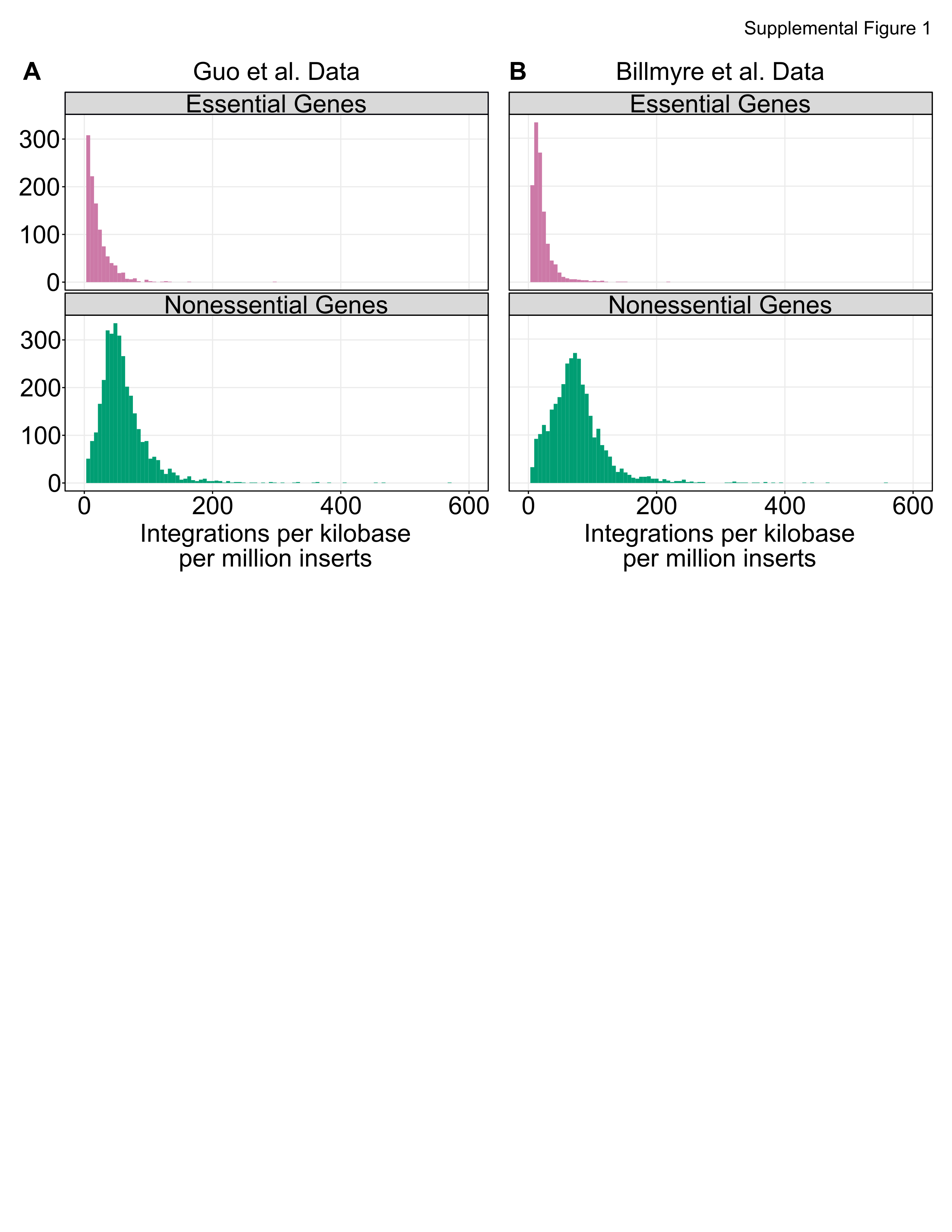

Supplement: S1 Fig — Histograms of insert density per gene both from published data [30] (A) and our data (B). Both plots are normalized with the number of unique insertion sites per gene normalized to the length in kilobases and per one million inserts. Genes annotated essential in Guo et al. [30] are in pink (top) and genes annotated nonessential are in green (bottom). (TIF) [file pgen.1010462.s001.tif]

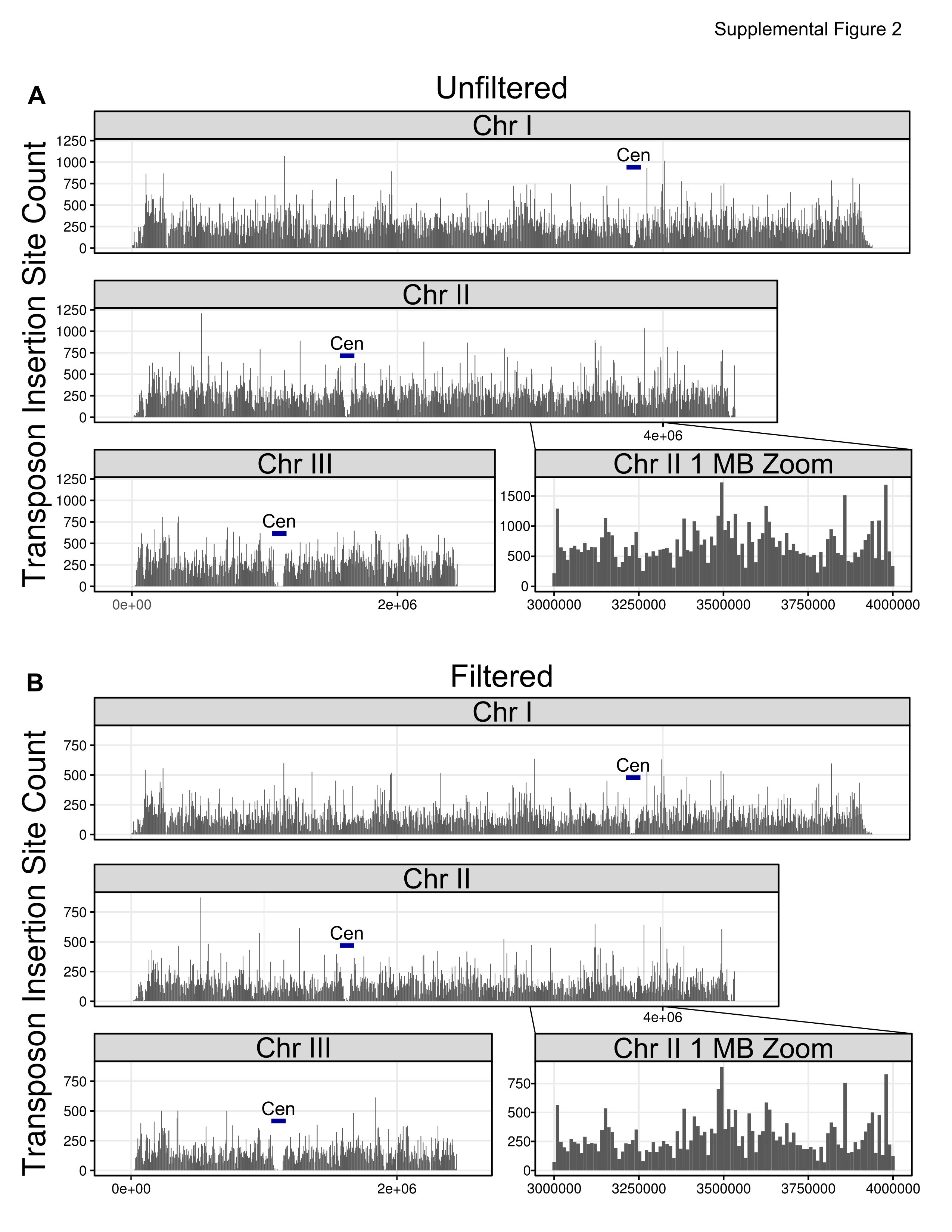

Supplement: S2 Fig — Genome-wide histograms of unique insert density before (A) and after (B) filtering to remove inserts with fewer than 8 unique ligation products in our sequencing. A 1 megabase region of chromosome II is shown as an inset. While filtering reduces the total number of inserts in a window, it does not substantially change the relative insert density. (TIF) [file pgen.1010462.s002.tif]

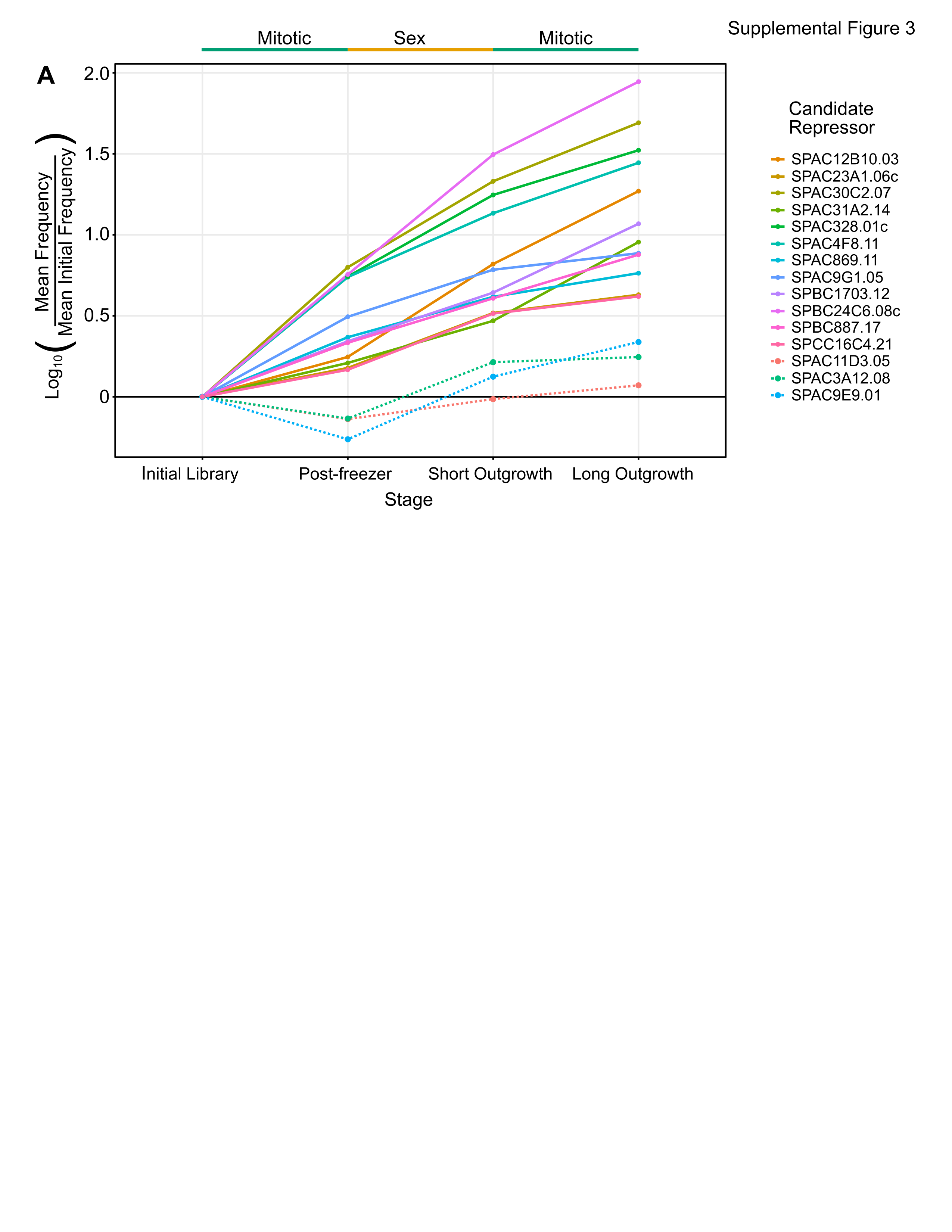

Supplement: S3 Fig — TN-seq insert frequencies for 15 genes identified as candidate repressors of sexual reproduction. The y-axis displays the log10 adjusted ratio of mean insert frequency across a gene to the mean insert frequency for that gene at the first sequencing step. The first and third segments are vegetative growth, while the second is a sexual reproduction step. Genes whose insert frequencies did not increase at every step are shown with dashed lines. (TIF) [file pgen.1010462.s003.tif]

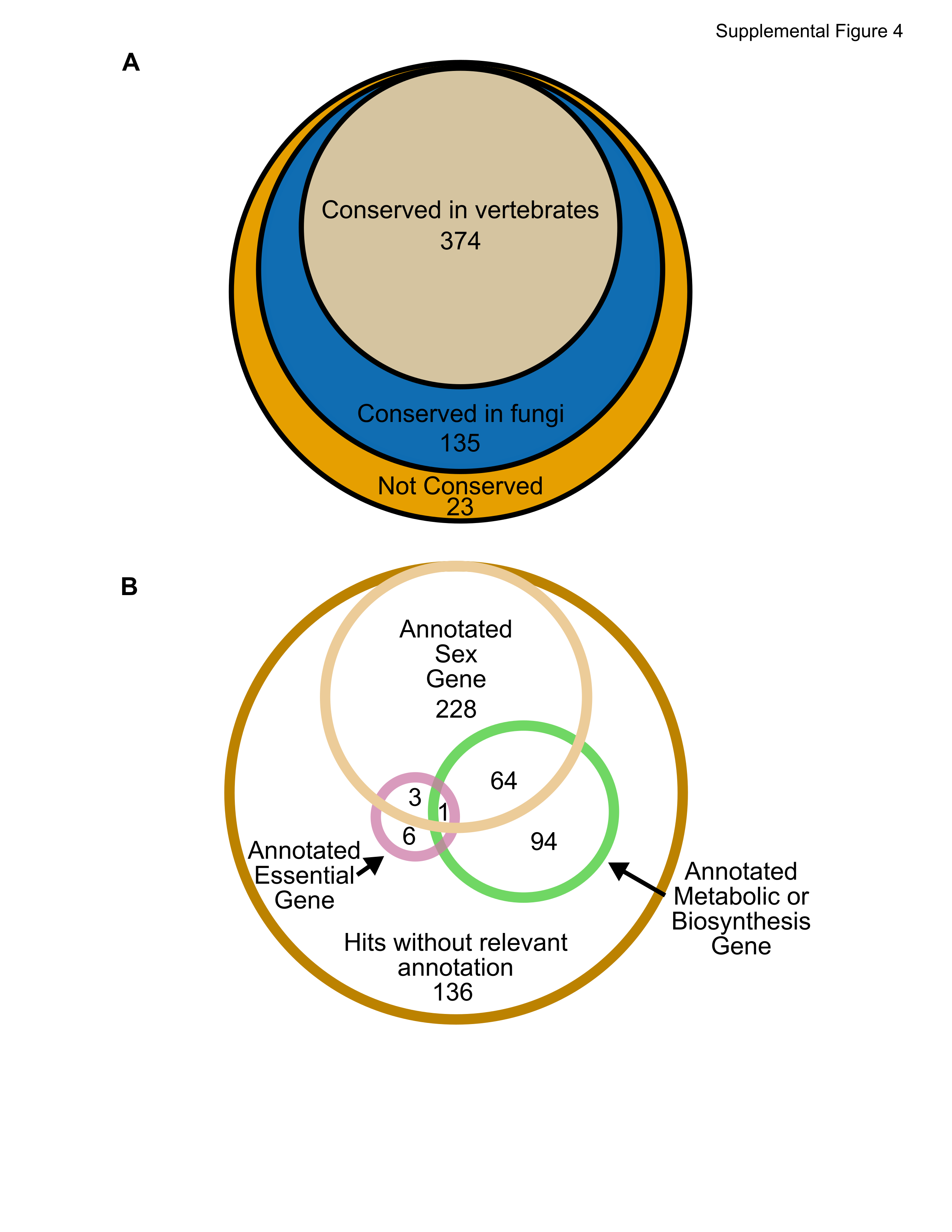

Supplement: S4 Fig — Venn diagrams showing the breakdown of candidate genes by conservation status (A) and by function (B). Both gene sets are broken down in S1 Table and both are derived from annotations on Pombase [66]. (TIF) [file pgen.1010462.s004.tif]

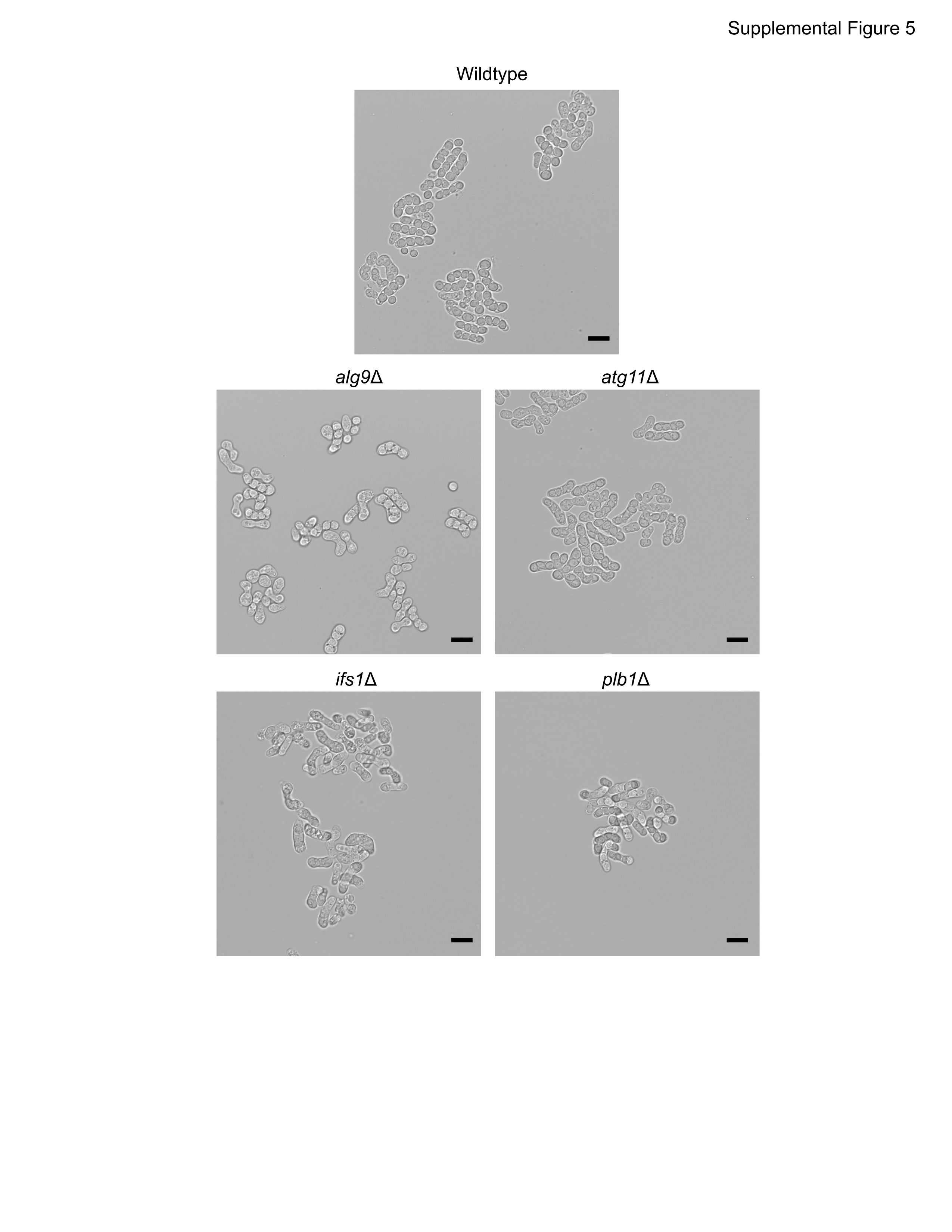

Supplement: S5 Fig — Imaging on an AXIO Observer.Z1 (Zeiss) wide-field microscope with a 40x C-Apochromat (1.2 NA) water-immersion objective of wild type and mutant S. pombe sexual spores/asci produced after 2 days at 25°C on MEA. Scale bars indicate 10 microns. (TIF) [file pgen.1010462.s005.tif]

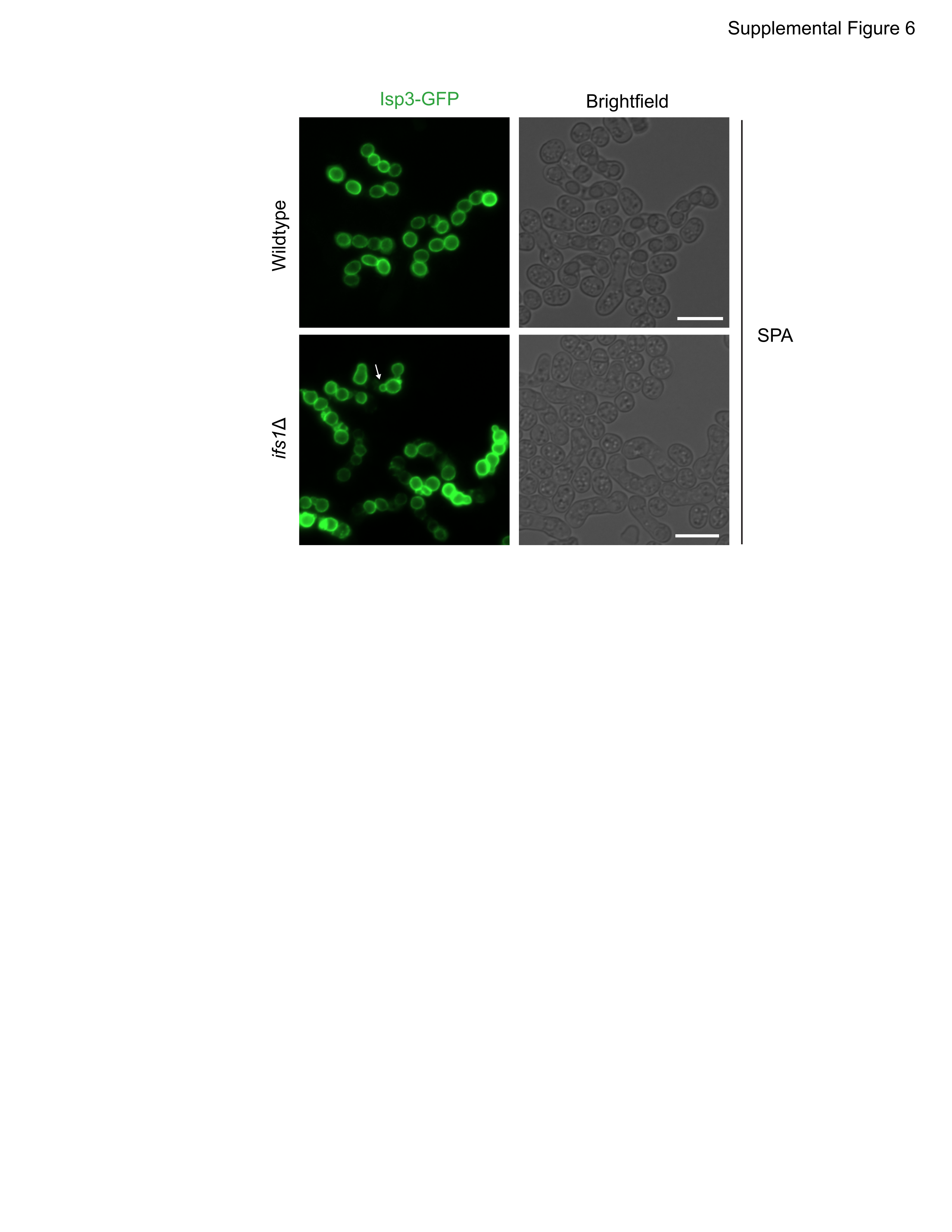

Supplement: S6 Fig — Isp3-GFP was visualized in wild type and ifs1Δ mutants on an AXIO Observer.Z1 (Zeiss) wide-field microscope with a 40x C-Apochromat (1.2 NA) water-immersion objective after incubation for 2 days at 25°C on SPA medium. Scale bars indicate 10 microns. Arrow indicates a “snowman” spore. (TIF) [file pgen.1010462.s006.tif]

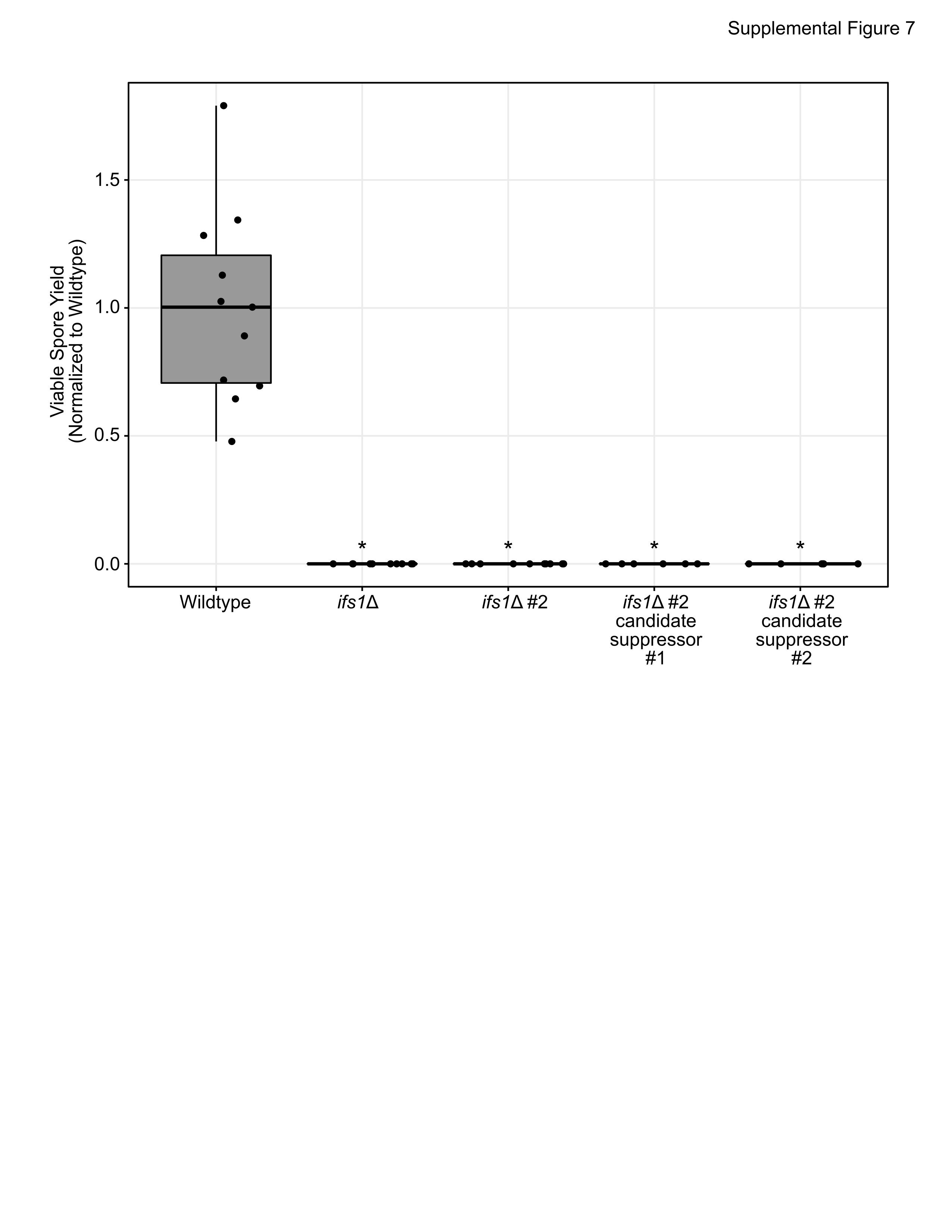

Supplement: S7 Fig — Viable spore yield assay showing on the y-axis the number of spores produced per yeast cell plated, normalized to the mean value for wild type. Cells were incubated on MEA plates in a dense growth spot for 3 days at 25°C prior to spore isolation. Results from two independent ifs1Δ mutants are displayed, as well as from two spores that germinated from independent biological replicates of the ifs1Δ #2 mutant. All mutants were assayed in a set of at least 5 biological replicates alongside at least 5 wild type replicates. Points display results from a single replicate, normalized to the mean from the corresponding wild type controls. The boxplots summarize the underlying points and show first quartile, median, third quartile while the whiskers show the range of the data to a maximum of 1.5 times the interquartile range below and above the first and third quartile, respectively. Points outside the whiskers can be considered outliers. (TIF) [file pgen.1010462.s007.tif]

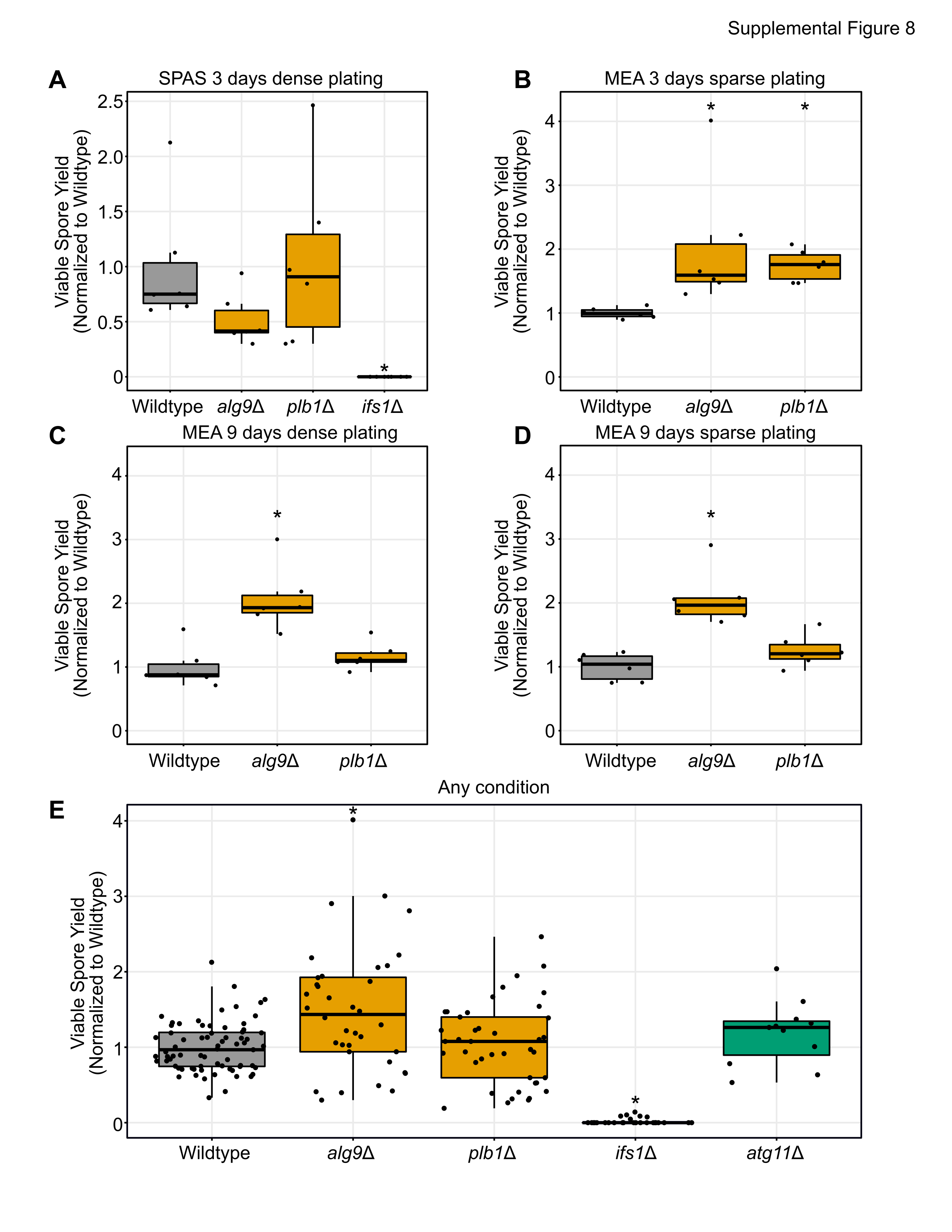

Supplement: S8 Fig — A-E) Viable spore yield assay showing on the y-axis the number of spores produced per yeast cell plated, normalized to the mean value for wild type. Points display normalized results from a single replicate. The boxplot summarizes the underlying points and show first quartile, median, third quartile while the whiskers show the range of the data to a maximum of 1.5 times the interquartile range below and above the first and third quartile, respectively. Points outside the whiskers can be considered outliers. A) Cells incubated on SPAS plates for three days at 25°C in dense cell patches (Mann-Whitney U test, alg9Δ, p = 0.065; plb1Δ, p = 0.94; ifs1Δ, p = 0.0044). B) Cells incubated on MEA plates for three days at 25°C at low density as in the original TN-seq assay (Mann-Whitney U test, alg9Δ, p = 0.0022; plb1Δ, p = 0.0022). C) Cells incubated on MEA plates for nine days at 25°C in dense cell patches. (Mann-Whitney U test, alg9Δ, p = 0.0043; plb1Δ, p = 0.18) D) Cells incubated on MEA plates for nine days at 25°C at low density. These conditions match the original TN-seq assay, except that spores were not germinated in liquid. (Mann-Whitney U test, alg9Δ, p = 0.0022; plb1Δ, p = 0.24), E) Summary data encompassing all conditions tested for these three mutants. This includes data from Fig 3D as well as S8A–S8D Fig (Mann-Whitney U test, alg9Δ, p = 0.0013; plb1Δ, p = 0.73; ifs1Δ, p = 1.1*10−14; atg11Δ, p = 0.14). (TIF) [file pgen.1010462.s008.tif]

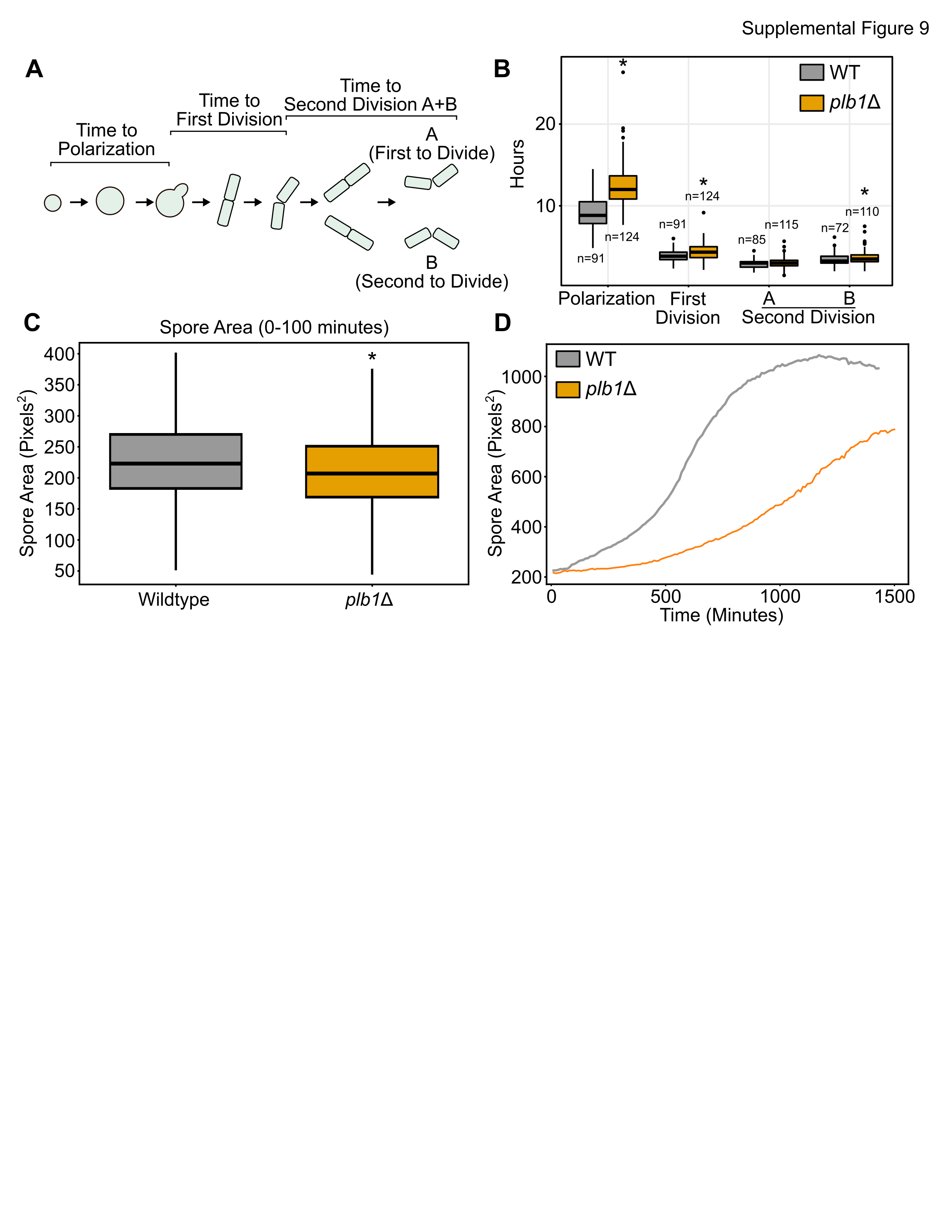

Supplement: S9 Fig — A) S. pombe spores undergo several landmarks in the process of germinating. Spores initially begin growing isotropically (ie. swelling). This phase ends when cells begin polarized growth and elongate on one side. This cell will eventually divide by fission for the first time and each of those daughters will go on to divide a second time after some delay. We scored each of these landmarks manually from videos using Fiji. Unlike in our microfluidics approach, we were unable to score the delay to swelling or the third cell division with this approach. B) Spores were plated on YEA+SUP plates and a punch was immediately taken and imaged at 32°C for 24 to 48 hours. Videos of spore germination were scored and time between each step in spore germination was tracked for individual spores. The boxplot shows first quartile, median, and third quartile while the whiskers show the range of the data to a maximum of 1.5 times the interquartile range below and above the first and third quartile, respectively. Points outside the whiskers can be considered outliers. C) Histogram of spore sizes from the first 10 frames (100 minutes) of videos of spore germination. Wild type and plb1Δ are each derived from at least two videos each from two separate days. Spores were identified using deep learning (see methods). D) Plot of average cell area over the course of videos of spore germination. Spores were identified via deep learning. Data are derived from at least two videos each from two separate days. Average spore sizes stabilize once spores begin to divide and grow vegetatively as yeast cells. Data are truncated at 1500 minutes as dividing cells begin to affect the average in plb1Δ mutants. The average size of plb1Δ mutant cells does not reach wild type levels by the time cells have divided enough to make continued tracking impossible with this approach. (TIF) [file pgen.1010462.s009.tif]

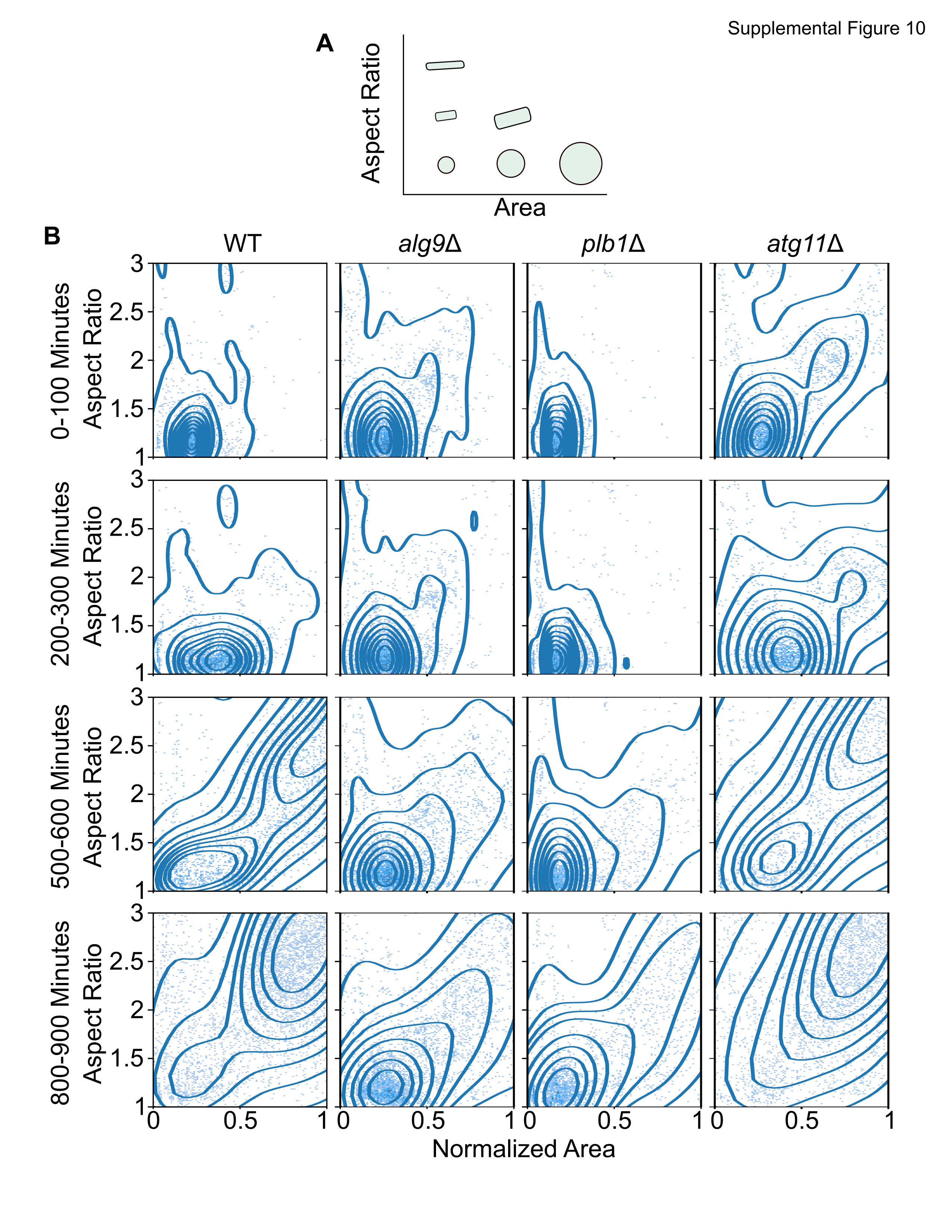

Supplement: S10 Fig — A) Schematic of plots of aspect ratio versus area. The y-axis displays aspect ratio, where a minimum value indicates round cells and larger values indicate more oblong cells. The x-axis displays normalized cell size. As shown in the cartoon, a higher aspect ratio indicates cells that are more elongated, while a higher area indicates cells that are larger. B) Two-dimensional histograms showing the entire population of spores for wild type or mutant in a given time window. Contour lines are added to help visualize concentration of cells. (TIF) [file pgen.1010462.s010.tif]

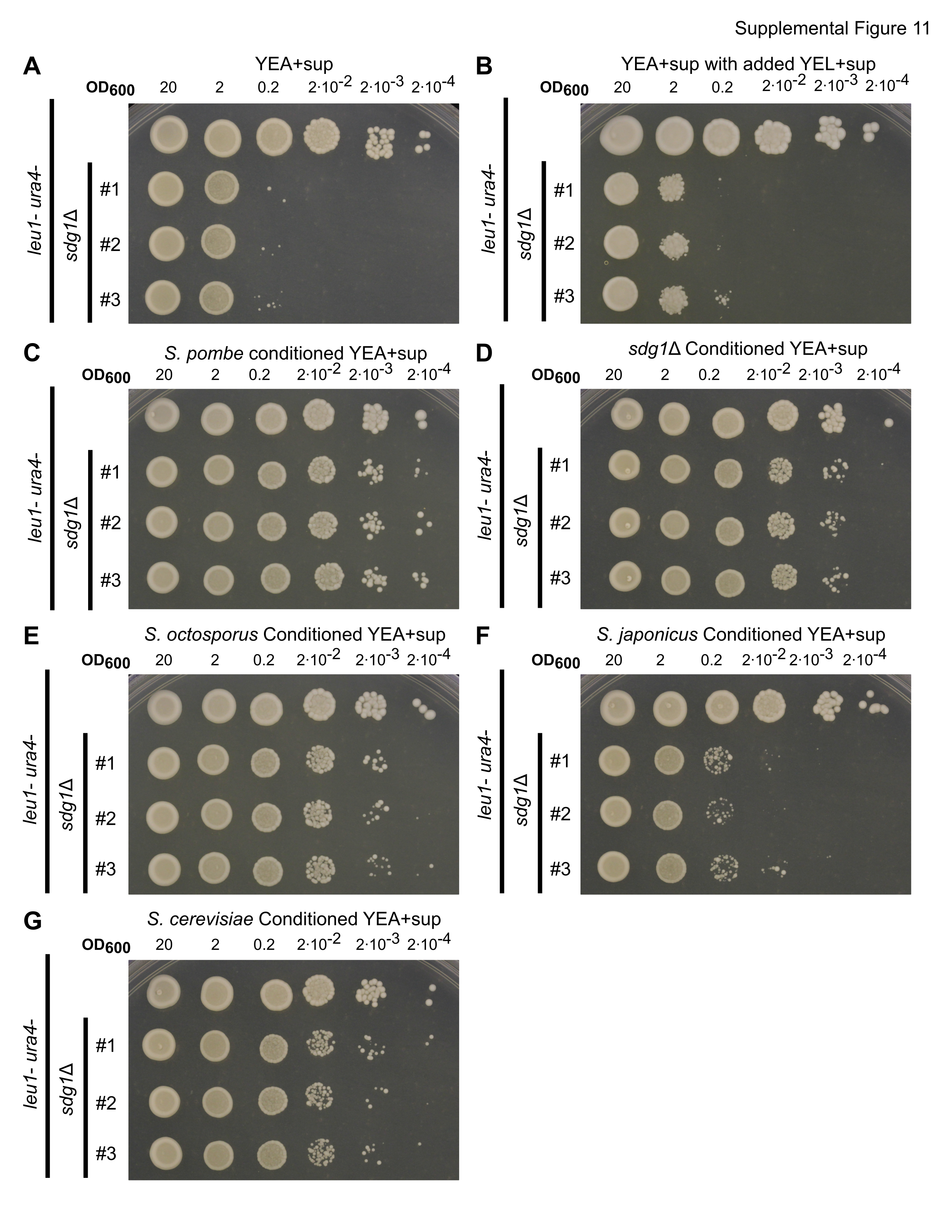

Supplement: S11 Fig — A-G) Spot dilution assays with 5 μL spots plated. The initial leftmost spot is of OD600 = 20 culture and each successive spot is a 10-fold dilution, so that the final spot should be 105 less concentrated than the first. All four experiments were conducted on the same day with the same dilution series of parent strain (ura4-D18, leu1-32) and three independent sdg1Δ mutants on the same genetic background (sdg1Δ::kanMX4, ura4-D18, leu1-32). All assays were also incubated for 4 days at 32°C. A) Spotted to standard yeast extract agar (YEA+SUP). B) Spotted to YEA+SUP where half the water had instead been replaced with yeast extract liquid with supplements (YEL+SUP) medium as a control for conditioned medium. C) Spotted to conditioned YEA+SUP medium where half the water had instead been replaced with YEL+SUP pregrown with the parent strain S. pombe (ura4-D18, leu1-32) (see methods). D) Spotted to conditioned YEA+SUP medium where half the water had been replaced with YEL+SUP pregrown with an sdg1Δ mutant (sdg1Δ::kanMX4, ura4-D18, leu1-32). E) Spotted to conditioned YEA+SUP medium where half the water had been replaced with YEL+SUP pregrown with S. octosporus. F) Spotted to conditioned YEA+SUP medium where half the water had been replaced with YEL+SUP pregrown with S. japonicus. G) Spotted to conditioned YEA+SUP medium where half the water had been replaced with YEL+SUP pregrown with S. cerevisiae. (TIF) [file pgen.1010462.s011.tif]

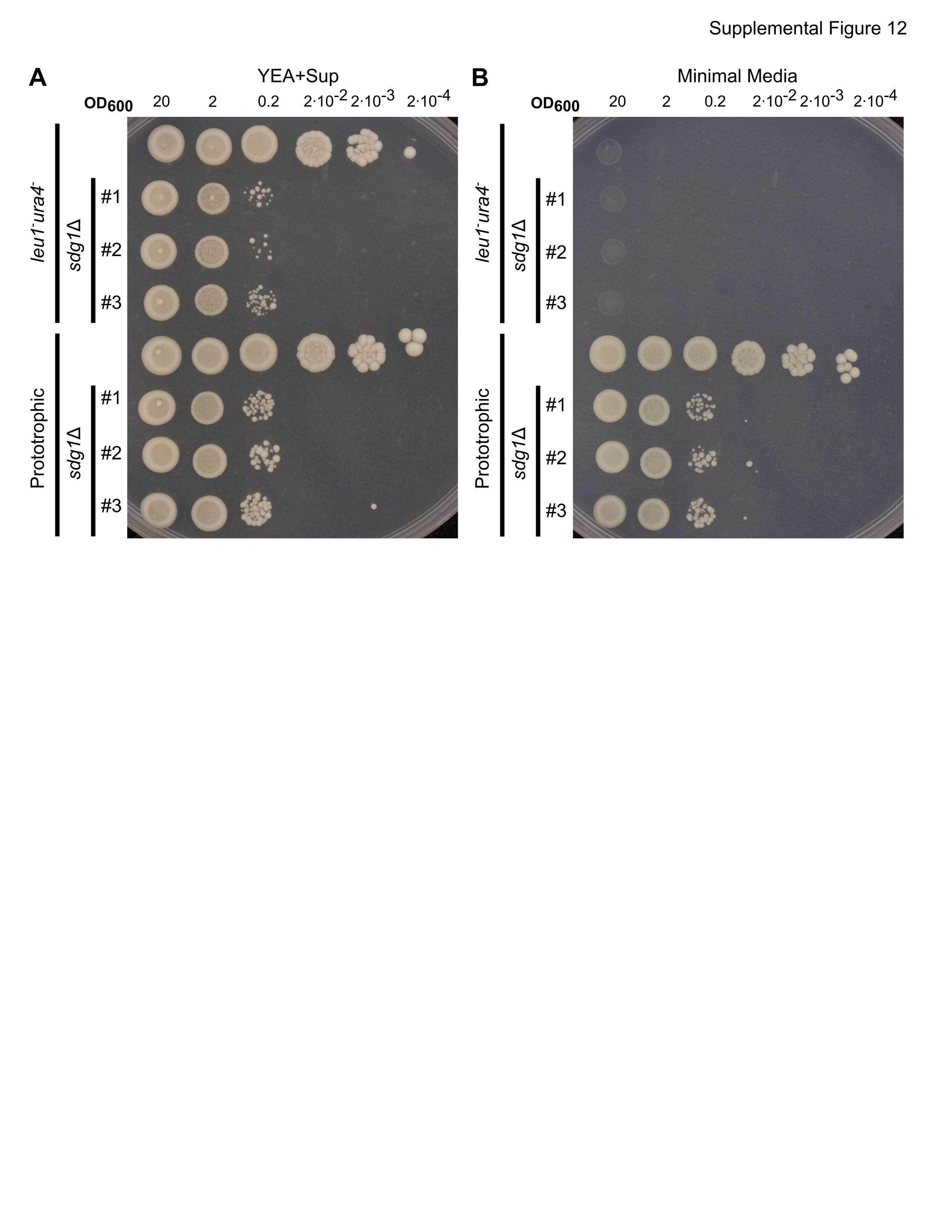

Supplement: S12 Fig — A-B) Spot dilution assays with 5 μL spots plated. The initial leftmost spot is of OD600 = 20 culture and each successive spot is a 10-fold dilution, so that the final spot should be 105 less concentrated than the first. Both experiments were conducted on the same day with the same dilution series of parent strain (ura4-D18, leu1-32), three independent sdg1Δ mutants on the same genetic background (sdg1Δ::kanMX4, ura4-D18, leu1-32), a wild type prototroph (h90), and three independent prototrophic sdg1Δ mutants. A) Spotted to standard yeast extract agar (YEA+SUP) and incubated for 4 days at 32°C. Note that the top half of this panel is the same experiment presented in Fig 5C. B) Spotted to minimal medium and incubated for 4 days at 32°C. (TIF) [file pgen.1010462.s012.tif]

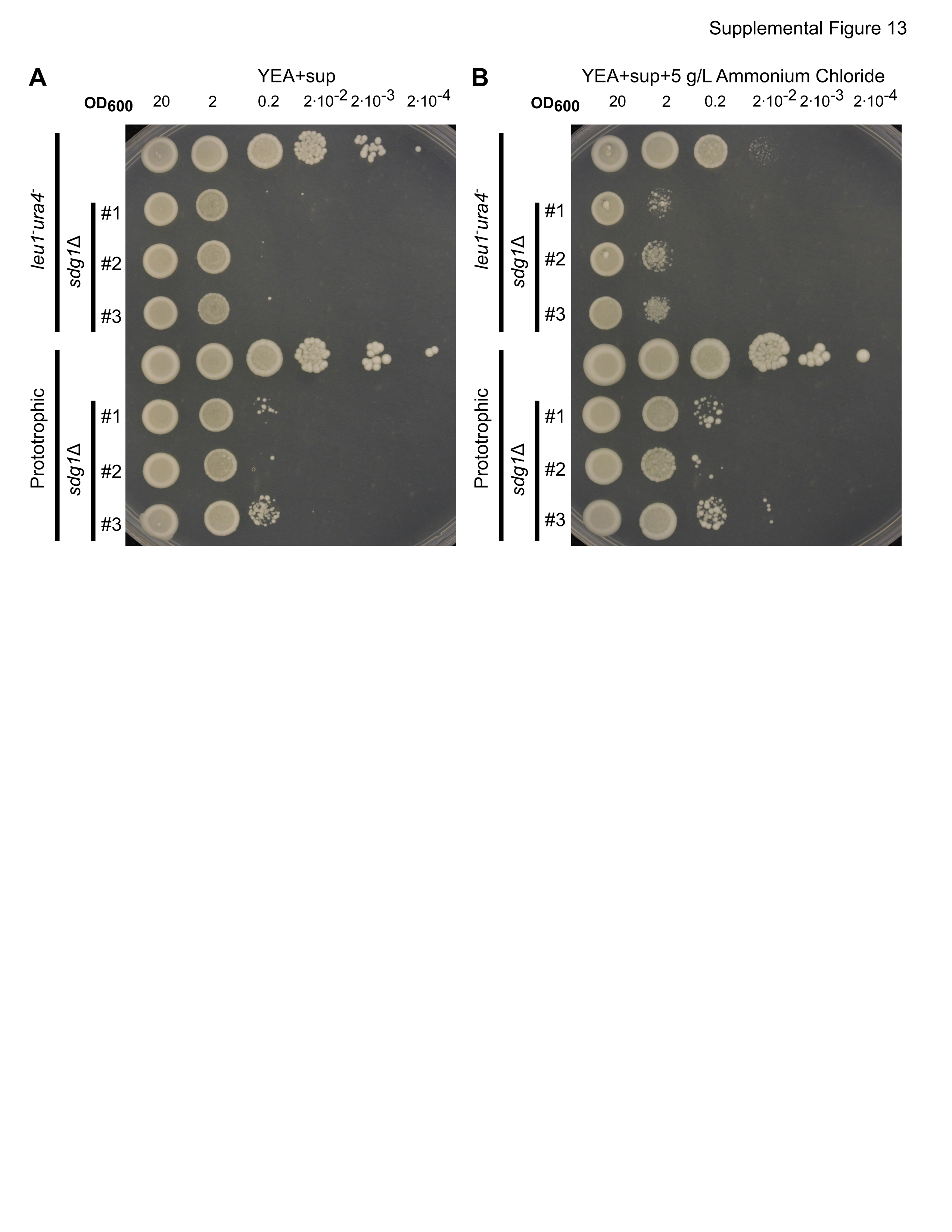

Supplement: S13 Fig — A-B) Spot dilution assays with 5 μL spots plated. The initial leftmost spot is of OD600 = 20 culture and each successive spot is a 10-fold dilution, so that the final spot should be 105 less concentrated than the first. Both experiments were conducted on the same day with the same dilution series of parent strain (ura4-D18, leu1-32), three independent sdg1Δ mutants on the same genetic background (sdg1Δ::kanMX4, ura4-D18, leu1-32), a wild type prototroph, and three independent prototrophic sdg1Δ mutants. A) Spotted to standard yeast extract agar (YEA+SUP) and incubated for 4 days at 32°C. B) Spotted to yeast extract agar with 5 g/L supplemental ammonium chloride and incubated for 4 days at 32°C. (TIF) [file pgen.1010462.s013.tif]

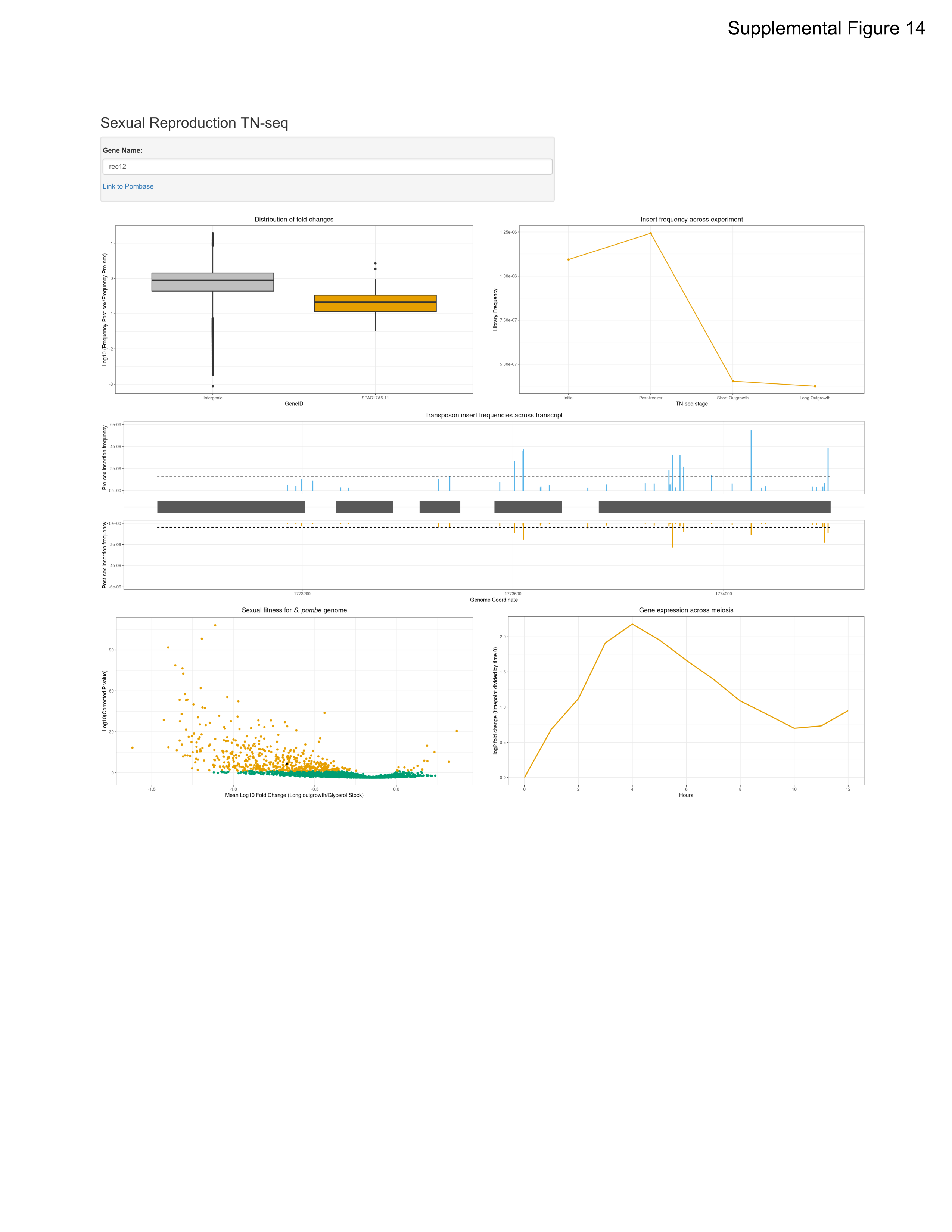

Supplement: S14 Fig — Screenshot of a publicly available interactive Shiny app that visualizes data from the TN-seq assay. There are five plots, displaying the distribution of insert fold changes within a gene (as in Fig 2B), the mean insert frequency within a gene over time (as in S3 Fig), the site-by-site frequency across a gene (as in Fig 2A), a volcano plot summarizing the entire experiment (as in Fig 2C), and a plot of transcription throughout meiosis for that gene from the Mata et al. dataset [49] (as in Fig 2D). This app will accept S. pombe gene names, either as systematic names or common names. (TIF) [file pgen.1010462.s014.tif]

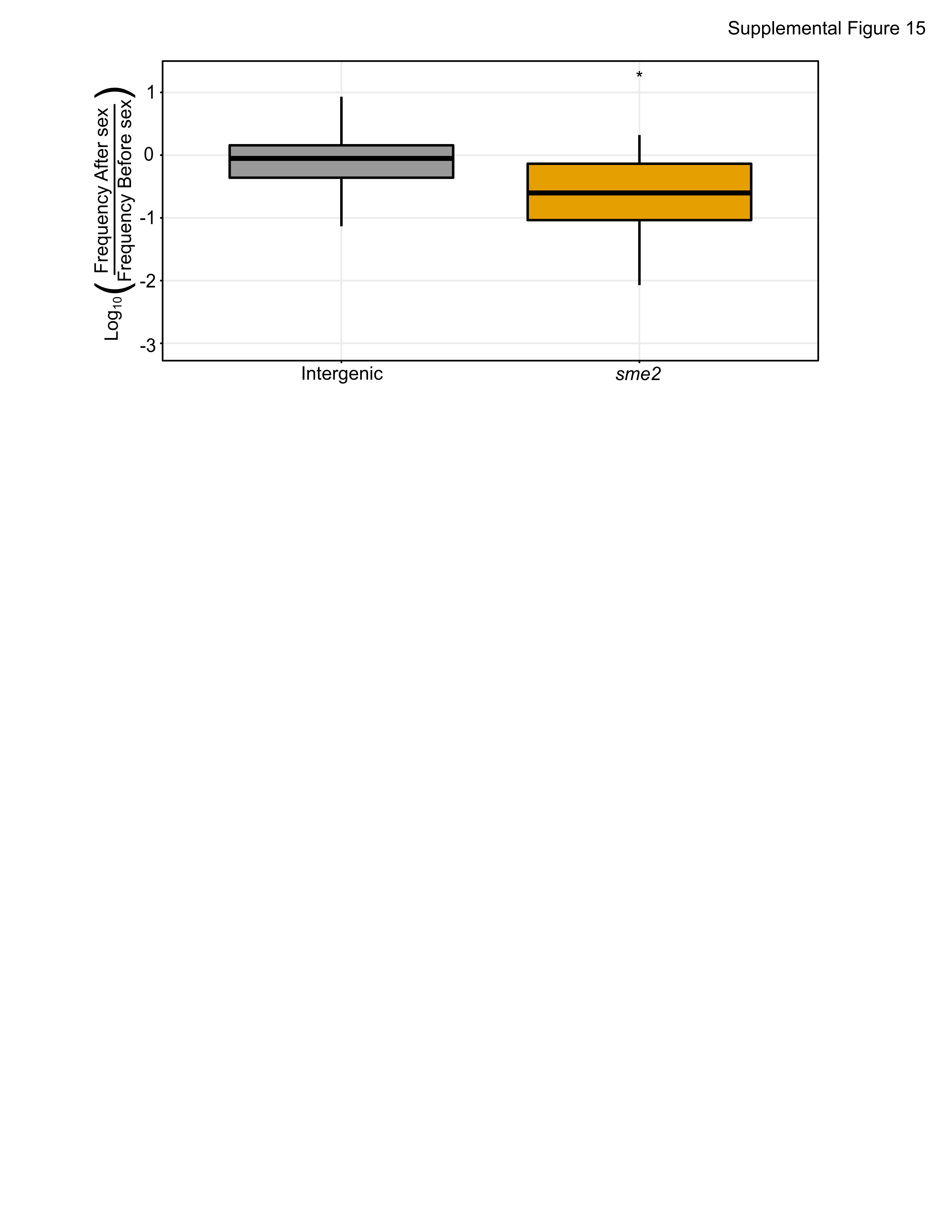

Supplement: S15 Fig — Boxplot displaying distribution of log10-adjusted fold changes in insert density after sex (ie. frequency after sex/frequency before sex). Boxplots show first quartile, median, third quartile. The whiskers show the range to a maximum of 1.5 times the interquartile range above and below the first and third quartile, respectively. Outlier data points (outside the whiskers) are not displayed. This results in 5,716 of 235,578 intergenic sites, and 0 of 78 sites from sme2 not being displayed although those data were considered in the statistical analyses. Inserts in intergenic regions are indicated in grey and inserts into the known meiotic noncoding RNA sme2 are shown in orange. (TIF) [file pgen.1010462.s015.tif]
