# Supplementary material for: Genome-wide quantification of contributions to sexual fitness identifies genes required for spore viability and health in fission yeast
Source: PLoS Genet. 2022 Oct 27;18(10):e1010462. doi: 10.1371/journal.pgen.1010462 (PMC9668190; doi:10.1371/journal.pgen.1010462)
Supplement: Attachment — Submitted filename: PLOS Genetics response letter.docx [file pgen.1010462.s023.docx]

We thank the reviewers for their time and attention to detail in reviewing our manuscript and for providing constructive feedback. Although the paper was not appropriate for *PLOS Biology*, we think it is a good match for the more specialized *PLOS Genetics* audience. Here we respond to each of the individual comments and concerns that arose during the review process. In our response we include both a tracked-changes and a clean copy of the manuscript. Below we have endeavored to address all of the review comments point by point.

REVIEWERS' COMMENTS:


Reviewer #1:

Review of Genome-wide quantification of contributions to sexual fitness identifies genes required for spore viability and health in fission yeast
By R. Blake Billmyre et al.

Understanding developmental and cell-differentiation type responses in genetically amenable model eukaryotes is a powerful and relevant endeavor. Many studies of mating, filamentous growth, and sporulation in budding and fission yeasts have been fruitful in identifying regulatory pathways and new biology. Here, the authors use a powerful and comprehensive transposon approach to yield an unexpectedly large number of genes that impact mating-> sporulation->germination pathways in S. pombe. In addition to the number of genes identified, the approach yielded new phenotypes that have been understudied and underappreciated, and the authors develop new approaches to explore these new phenotypes.

The main concern here is the claim about the novelty of the approach as a method. Transposon screens, albeit extraordinarily powerful, have been widely utilized, including in S. pombe. High-throughput sequencing has become routine, even by using bar coding. What is novel about this study is not the technique. Rather, the novelty of the study comes from the large number of new genes identified that regulate mating, switching, sporulation and/or germination. Also novel were the new phenotypes that were classified, including germination health, and the 'low density' growth defect phenotypes that were identified. The study was comprehensive and thorough.

A second issue is that the study does not get to the bottom of any one gene's role in regulating a biological process. The four genes on which the study was focused did not reveal any sort of mechanistic connection to signaling, meiosis, or spore function. How loss of the social distancing gene leads to dependency on conditioned media remains enigmatic.

There is a lot to like in this paper. The paper was well written, clear, and interesting. The authors did a good job of acknowledging the limitations of their screen. New techniques were developed, including competition assays to evaluate spore germination and a microfluidics approach to examine germination delay. Machine learning algorithms were employed, and statistical analyses were performed throughout. An app was developed to help researchers navigate the authors' data.

In addition, there are some issues that should be addressed to clarify or improve the study.

* Was the sequencing performed to insure that second-site mutations were not responsible for the phenotypes generated? Or were the phenotypes independently confirmed? It looks as if only transposon-associated regions were sequenced. This concern is somewhat alleviated by the fact that multiple hits were identified in many genes. And four genes on which the study is focused were independently deleted and the phenotypes verified.

As noted by the reviewer above, this study applies a previously developed transposon sequencing approach, which has previously been used broadly in bacteria and in a few fungi, including *S. pombe*. This approach does rely on sequencing only the boundary between the transposon insertion and the genome to enable cost-effective mapping of the extremely high transposon insertion density. We applied an existing Hermes transposon/*S. pombe* system to generate transposon mutants, which was developed by the Levin lab and has previously been demonstrated to achieve majority single transposon insertions (PMID: [17947404](https://pubmed.ncbi.nlm.nih.gov/17947404) from the Levin lab (also reviewer #3)). We now cite this in the results section and describe this previous finding.

Further, a strength of our assay achieving high insert depth is that multiple insertions across a gene reduce the impact of a single cell having multiple inserts. Any two genes are highly unlikely to share numerous co-insertions, meaning that an erroneous signal at one site should be swamped out by other insert sites in a gene. As the reviewer noted, our success in validating the phenotypes observed demonstrates the reliability of our method as well.

* Transposons might interfere with (but not fully compromise) essential gene function by their proximity to essential genes. Was this taken into account for this study?

We thank the reviewer for this suggestion. We did limit the definition of a gene to the region between the ATG and stop codon, excluding UTR and promoter regions that are closer to neighboring genes. Further, our assay tests the change in frequency between a starting vegetative culture and a final post sexual reproduction culture and uses the average fold change across multiple inserts in a single gene. As a result, the signal from an insert particularly close to the 5’ or 3’ end of a gene and thus closer to a neighboring essential gene is likely to be swamped out by more distant inserts with less or no effect or that neighboring gene.

Analysis of the hits and non-hits from our dataset shows that 40% of non-hits are next to at least one essential gene and 45% of hits are next to at least one essential gene. This difference is statistically significant (p=0.0184, Fisher’s Exact test) however the magnitude of the change suggests that if there is enrichment for neighboring essential genes in our hits, it is unlikely to explain a large portion of them. Further, this potential issue is not unique to transposon-based screens and neighbor effects exist in deletion collection screens as well. Once again, our validated genes did not encounter any cases where neighboring essential genes explained the phenotypes.

* The term h90 is not clear to the non-expert.

We thank the reviewer for this comment and have altered the introduction of h90 to clarify- it simply means a strain is wildtype for the mating type locus and capable of switching mating types.

* The authors claim that "Building a library of cells with independent transposon insertions allows mapping of essential (pink) and non-essential (green) regions of the genome by measuring insertion density via sequencing." Did they do this? How closely did their screen overlap with known essentiality in this organism?

We did not use our data to re-predict essential genes, because our TN-seq assay was carried out using the same previously published approach from the Levin lab, who did use TN-seq and the same transposon to analyze essential genes (PMID: 23893486). Comparing our data to the annotated essential gene set showed a clear enrichment for inserts in non-essential genes vs essential genes (p < 1 * 10^-16^, Mann Whitney U test). In addition, plotting transposon insertion density normalized by gene length revealed a similar insert distribution to that seen by the Levin lab as well (below). We have added this figure to the text as Supplemental Figure 1.

* "The genes predicted to repress sex revealed a second complication." Is this correct as written? Genes could be involved in many processes related to overall reproduction.

We are not completely sure of the reviewer’s meaning in this comment but this section of the manuscript is intended to highlight a potential shortcoming in our approach. Specifically, we describe that fast or slow growing strains could imitate a sexual reproduction phenotype. One could thus imagine that they may give a false positive simply because, as the reviewer describes it, they are involved in a process related to overall reproduction. We have edited this section to make it more clear.

* In the paragraph starting with: "Our data is consistent with previous studies of sexual reproduction in S. pombe." It would be useful to know what fraction of genes known to be involved in mating, switching, and sporulation were identified. Were every major regulator of these pathways identified? Who was missed and why?

We thank the reviewer for this comment and agree that this is an important issue. As a result, we do have an in-depth and nuanced discussion of this in the discussion of the paper. To summarize, there is some overlap between the existing screens (over half of our identified genes had existing annotations related to sexual reproduction) but no single screen previously looked at the same end point that we used here. Some of those phenotypes, such as the relatively mild chromosome segregation defects identified by the Marston lab, will have relatively little impact on spore production and thus have little impact in our assay, despite having sexual reproduction annotations.

* Seems there is a typo here: "To better visualize ifs1Δ mutant spores, we generated a deletion of ifs1" I think it should be 'deletion of the IFS1 gene"

We appreciate the reviewer’s comment, but the use of the lowercase and italic *ifs1* is the correct nomenclature for *S. pombe*, while the reviewer’s text would be the correct nomenclature to describe *S. cerevisiae*.

* Why was spore germination not examined in panel 3F for ifs1?

Spore germination was not examined in this panel because *ifs1*Δ mutant spores germinated at an extremely low rate (see Figure 3D) and achieving similar data for *ifs1*Δ mutant spores to the other mutants was impractical. We have added text to explain this.

* Table 1 is confusing - why are there three wild types? If this data is 'negative data' perhaps it should be moved to the supplement.

Table 1 described 3 pairs of experiments conducted on different days, with each pairing a mutant with a wildtype control strain conducted on that same day. Pooling the wildtype data also results in aneuploidy rates that are not statistically significantly different between any of the mutants and wildtype. We agree with the reviewer that this data could be moved to the supplement and have done so as Table S2. This also includes a more detailed figure legend to address the reviewer’s confusion.


Reviewer #2:

This manuscript from Billmyre et al. describes a transposon mutagenesis/sequencing approach to screen for genes required for sexual development in fission yeast. The approach, which monitors the frequency of a given insertion in the population after cells undergo mating and meiosis, casts a wider net than earlier efforts at systematic screens, which is likely one reason that the authors identify over 500 genes that influence this process. The authors demonstrate unique phenotypes for a handful of these genes, including both known genes not previously implicated in mating and meiosis and genes not previously characterized.

The experiments described here are of very high quality. The statistical tests used are appropriate, the supplementary information provides important data on characterization of some of the mutants identified, and the methods are explained in sufficient detail to allow others to use this technique. Moreover, the dataset will likely be of useful resource to the S. pombe community and the sdg1 gene identified as required for growth at low population density is particularly interesting.

My only concern is whether these results are of broad enough interest for PLoS Biology. The transposon mutagenesis technique has been previously used in several organisms, including S. pombe. Though particularly well used here, this does not represent a significant methodological advance. Further, though many of the genes identified have homologs in other organisms, it is not clear, based on results presented here, whether there is reason to expect that many of these genes are involved generally in sexual reproduction in other organisms.

We thank the reviewer for this generally positive review. We believe that the findings should be of interest to others in investigating sexual reproduction in their organism of choice as well as to drive further studies in fission yeast. Testing homologs of the genes identified here in other species is beyond the scope of the work presented here.


Reviewer #3:
[identifies himself as Henry L Levin - see attachment for formatted version]

Review for Plos Biology paper (Billmyre et al., 2022):

Summary: In this manuscript, Billmyre et al identified multiple genes important for sporulation and germination using dense profiles of Hermes integration in S. pombe. Among the list of candidate genes, over half of them were annotated as sexual reproduction genes found by previous studies. This suggests the Tn-seq worked as a powerful tool to screen genes required for sexual reproduction. To note, they focused on three candidate genes (ifs1, plb1 and alg9) which were not discovered in previous screenings. Their assays found that ifs1 is important for spore viability and the others play key role in the initial stage of spore germination. Furthermore, the authors found the sdg1 contributes to cellular growth in low densities.

In all, the authors present some important and interesting findings of new genes required for yeast sexual reproduction. However, a couple of issues should be clarified before this paper can provide a significant impact on the field. For the interest of wide audience, the authors should provide more information of sporulation and germination in the introduction. Authors should consider the work of Tsuyuzaki et al (PMID: 32152323) as they investigated transcriptome during germination through scRNA-seq. Indeed, the reviewer found two genes sgo2 and cyp4 are present in both your Tn-seq hits and Tsuyuzaki's list of variable genes for dormancy breaking.

We thank the reviewer for this thorough and positive review. We agree that the paper would benefit from more information on sporulation and germination and have added this into the introduction. We have also added a comparison with the genes from Tsuyizaki et al to the discussion.

comments:


1. Line 104, a comprehensive review to reference is PMID: 32886545.

We thank the reviewer for this suggestion and have added the citation.

2. Line 110. Every gene is an over interpretation. Perhaps comprehensive would be a better word. Small genes are not readily identified, and functional RNAs are not readily identified.

We thank the reviewer and agree. We have edited the text accordingly.

3. Line 170 and throughout. This language is confusing. These are not eight independent insertion events occurring at the same nucleotide site. I believe the authors refer to their random barcode in the linker used in PCR amplification. These barcode counts are not necessarily the result of independent insertion events. It really reflects the number of progeny cells derived from the original insertion event. This is a reasonable approach to avoid jackpot PCR of single positions. But it should be better explained as monitoring the depth or representation of each insertion event in the library. The reader must understand the difference between independent events vs what the authors call inserts. A better word is required thoughout the manuscript, perhaps insertion reads or ligation events.

We agree with the reviewer that our language here is imprecise and potentially confusing. We have edited throughout accordingly by using the term “unique ligation product” to describe reads with a unique barcode rather than unique insert.

4. Line 184, the reviewer doesn't understand the point of "set the minimum detected value for any given insert site to one read" since the authors already filtered out the insertion sites with less than 8 reads.

We thank the reviewer for this question. We removed only sites with less than 0 reads in the starting sequencing pool, not those with 0 reads in selection conditions. As a result, multiple sites may have had, for example, 10 reads in the starting condition, but 0 in one of the later selection conditions. In order to calculate log ratios for these sites (eg. log(0/10)) we changed the 0 values to 1 (eg. log(1/10)). This has the effect of damping signals, but it enables the calculation of log ratios at the sites with the strongest effects (because log(0/10) is undefined but log (1/10) is not). We have added brief text to make this reasoning more clear.

5. Line 213, the authors could make Venn diagrams to specify how many candidate genes are conserved in fungi and in vertebrate and how many candidates are overlapped with the factors identified in the previous screenings.

We thank the reviewer for the suggestion and have added Venn diagrams as Supplemental Figure 4.

6. Among the list of candidates, the reviewer noticed there are some Tn-seq hits matching to essential genes (i.e., exo70, iss1), but the authors didn't comment on these candidates. Some essential genes may tolerate Tn insertions at particular ORF locus without affecting the cell viability (ref 30).

We thank the reviewer for this comment. We have compared our list of hits to the pombase essential gene annotations. 10 of our hits, including *sdg1*, are annotated as essential genes. Four of these were specifically mentioned in the text, but we have now added additional text describing this in the results and an additional column to Supplemental Table 1 with the viability annotation from pombase.

7. Line 221, authors should explain how the p value was calculated in the method section. In the part of S.pombe functional annotation (Method), authors should include more details.

We thank the reviewer for catching this omission. In answering this question, we realized these tests were originally conducted using t-tests rather than the more appropriate Mann-Whitney U test for this type of data. We have recalculated p values using this test, which resulted in slight changes in p-values but no change in the finding. We have made this change to the text and added the requested description to the methods.

8. Line 260, the microscope pictures in Sup. Fig 2 needs scale bars.

We thank the reviewer for catching this oversight and have made this addition to the figure and the figure legend.

9. Line 397-403, the Fig number of Sup. Fig 6 and 7 should be switched.

We disagree with the reviewer here. We suspect the reviewer believes we referenced Supplemental Figure 7 before Supplemental Figure 6 (which we do in the line described), however, in the section immediately before Lines 397-403, we reference Supplemental Figure 6 A and B. We do not believe renumbering is appropriate.

10. In the method part, the authors should provide more details about how to construct the sdg1 deletion strain since the mutant is not viable under low density. Typically, it's impossible to obtain dense colonies when use drugs to select for positive clones.

This is an interesting question. We did not know when we generated our *sdg1* mutants that we were looking for a low-density growth defect and we were able to generate our mutants using a standard lithium acetate transformation protocol with no modifications. We speculate that the other non-transformed cells on the plates/the plated recovery medium provided enough quorum molecules to enable transformed cells to grow from single cells. We now mention this in the methods.

11. Line 670, please use "next-generation sequencing".

We thank the reviewer and have made the suggested change (next-gen sequencing to next-generation sequencing).

12. In the discussion, authors should note that 20 of the 532 Tn-seq candidate genes are related to the autophagy pathway. It may not be new that autophagy is required for the sporulation in yeast (PMID 33608896 and 19778961). But the authors could emphasize the atg genes from the Tn-seq hits act as additional evidence of the cross-talk between autophagy and sporulation in S.pombe.

We thank the reviewer for this suggestion and insight and have added text to highlight the autophagy gene hits.


Reviewer #4:

The manuscript entitled "Genome-wide quantification of contributions to sexual fitness identifies genes required for spore viability and health in fission yeast" describes a transposon-based screen to determine genes important for sexual reproduction in S. pombe. The approach expands on prior transposon screens by incorporating high throughput sequencing to rapidly identify genes with transposon insertions. By coupling this transposon mutagenesis and detection with assessment of reduced spore viability, the authors were able to identify several genes that likely affect sexual reproduction. The authors were able to show the utility of this approach by identifying a previously uncharacterized gene and a novel mutant phenotype and combining machine learning based image recognition to evaluate particular mutant phenotypes. The strength of the approach is that it does not require the large facilities and complicated strain backgrounds that are required to create a deletion collection or modify a deletion in a manner such as those approaches pioneered by Charlie Boone.

Overall the manuscript was well written, detailed, is of great interest to the fungal world and adds to the knowledge of genes that are involved in sexual reproduction. However, it is not clear this study makes a large enough advance, technically, in its utility to a broad number of organisms, or in its findings of genes that are involved in sexual reproduction, since many genes important in sexual reproduction have already been identified (in budding yeast for example) thus it seems that a more specialized journal would be more appropriate. In addition, one major concern is that although the approach was applied to finding genes that affect sexual reproduction, the specificity to sexual reproduction is not clear. In this study sexual reproduction would be affected if genes involved in mating, meiosis, spore development and proliferative growth. The fact that it is difficult to fully distinguish genes that affect proliferative growth cleanly from those that affect genes that are truly involved in sporulation. The assay also doesn't distinguish the genes that are involved in diploid formation from those involved once sporulation is entered since the assay starts from haploid cells rather than diploid cells.

We thank the reviewer for this review. We believe that one of the strengths of this approach is the ability to assay the entirety of sexual reproduction in a single experiment. We are not measuring specifically the ability to form diploids or for spores to germinate, although future experiments certainly could assay individual steps in the process (as described in the discussion). Instead, we measure contribution to the entire process. Notably, these steps are conserved across sexual reproduction, albeit sometimes in a different order (yeast sexual reproduction starts with formation of diploids while mammalian sexual reproduction is typically thought of as ending with formation of diploids).

Otherwise there are only a few additional minor concerns:

Line 169. It is important to show the insert coverage particularly since you are removing the low frequency inserts. One reason why transposon mutagenesis was not more frequently used in the past was that the coverage is less complete than deleting each gene. Did you evaluate whether there was a Poisson distribution of the sites and after the removal is there is no longer a Poisson distribution? With your approach, it seems you are further biasing the chromosomal regions to those genes that are easier for the transposon to insert into. Although it is pointed out that the average insert is 1 out of every 38 bases, it would be important to present a genomic map of inserts to see where there is a lack of coverage.

We understand the reviewers concern about non-random insertion but do not believe it is a significant issue here. Past use of the Hermes transposon has shown there is relatively little bias in insertion site (PMID: 23893486 for example) and we saw little evidence of insertion bias here. We have added text to the introduction to describe this.

The main driver of differences in number of transposon insertion sites per gene is the phenotypic impact of those insertions, as shown in the newly included Supplemental Figure 1 (shown and discussed in answers above). Genes annotated as essential on average have a lower transposon insertion frequency per gene length (0.6% of bases have an insertion on average) than nonessential (4.7% of bases have an insertion on average).

This means that the distribution of inserts per gene is not simply a distribution of random chance, but also the result of selection after that distribution. Neither the original data nor the filtered data is Poisson distributed based on a dispersion test, even if annotated essential genes are removed from the dataset.

In addition, our assay aims to control for transposon insertion frequency by comparing the final frequencies to the initial frequencies. Removing low frequency inserts increases our ability to do this accurately. As described in the text, this does reduce our power and we are only able to test for a role in sexual reproduction for approximately the same number of genes as the existing deletion collection.

However, there are no apparent differences in gross distribution of inserts across the *S. pombe* genome. As seen in the plot below (now included as Supplemental Figure 2), the number of insert sites obviously decreases but this does not result in dead spots in the genome, nor are there large dead spots in the genome in either condition outside of the repetitive centromere and subtelomere regions.

Line 711. More details about the plasmids containing the transposon and the transposase need to be provided. Which one is marked with ura+? One can eventually infer the information but it would be nice to actually provide it.

We thank the reviewer for noticing this oversight. While this information was present in Table S4 it has now been added directly to the methods section as requested.
