# Supplementary material for: Genome-wide quantification of contributions to sexual fitness identifies genes required for spore viability and health in fission yeast
Source: PLoS Genet. 2022 Oct 27;18(10):e1010462. doi: 10.1371/journal.pgen.1010462 (PMC9668190; doi:10.1371/journal.pgen.1010462)
Supplement: Attachment — Submitted filename: PLOS Genetics response letter.docx [file pgen.1010462.s024.docx]

We thank the reviewers for their time and attention to detail in reviewing our manuscript and for providing constructive feedback. Below we have endeavored to address all of the review comments point by point. In our response we include both a tracked-changes and a clean copy of the manuscript. We will note that the figures included in this submission appear lower resolution than the previous submission because of compression applied by the PLOS Genetics submission system. If the figures are difficult to see or interpret, the original uncompressed figure can be accessed via a download link on each page.

Reviewers Comments:

Reviewer #1: The manuscript by Billmyre et al. presents a Tn-Seq-based screen to identify genes in S. pombe required for wild-type sexual reproduction. The study identifies 532 such genes. The authors focus additional studies on four genes from the screen: ifs1, plb1, alg9, and sdg1. Deletion phenotypes of plb1, alg9, and sdg1 are unusual. plb1 and alg9 are required for wild-type spore health, as deletion phenotypes for these genes each indicate viable spores with low fitness. The sgd1 gene is reportedly required for growth at low cell densities.

Comments:

In total, the screen appears to have been implemented carefully, and it is nice to see that some phenotypes were confirmed by independent construction of deletion mutants. The remainder of the paper is less clear.

The paper would certainly be strengthened by providing some mechanistic insight regarding plb1, alg9, or sdg1; however, it will probably require significant effort to identify such mechanisms for these phenotypes – particularly for sdg1.

We thank the reviewer for this response. We agree that further investigation of the individual hits will likely be fruitful, but given the body of work already presented here, believe that this is outside the scope of this manuscript.

I think it would be helpful to indicate if any transposon insertion bias was observed in the mutagenesis screen from alignment of insertion sites. If findings for this mutagenesis are consistent with previous analysis of the Hermes transposon, it would be fine to reference a previous study.

We have used the same Hermes transposon plasmids used in previous *S. pombe* TN-seq experiments by other groups. Supplemental Figures 1 and 2 (added in the previous revision) show that the distribution of insert sites is similar between our initial library and the previously published experiments.

Further, our approach compares the frequency of transposon insertions at a set of constant sites before and after selection. The transposition happens only once, prior to any selection. Because of this comparative approach, even if insertional bias exists, it is of very limited impact. The one potential consequence is if transposons do not insert into a gene at all via insertional biases. We handled this in the existing manuscript by only assessing genes with multiple independent insertion sites (5 or more). As described, we were still able to score a similar number of genes to the deletion collection after this filtering.

I don’t have additional comments beyond the ones indicated by previous reviewers of the work.

Reviewer #2: This manuscript from Billmyre et al. describes genome-wide quantification about sexual differentiation of fission yeast using transposon mutagenesis with high-throughput sequencing. This is a very nice piece of work that contains huge amount of data. The results are mostly solid and well presented. Also, the manuscript is well written and will be interesting not only to yeast researchers but also general readers. Therefore, I recommend this manuscript for publication in PLOS genetics.
I have several minor comments as listed below.

In general, "wildtype" is written as "wild type".

We have made this change.

Line 334: "Plb1∆" should be "plb1∆".

We have made this change.

In the materials and methods section, the notation of the components of the medium is not unified. For example, in line 722, "Adenine" but in line 769, "ade".

We have unified the notation by preferring the full names of amino acid and nucleotide supplements throughout the materials and methods section.

Line 782: "ura+" is "ura-"?

The reviewer is correct and we have made this change.

Line 884: "glusalase" should be "glusulase".

We have made this change.

Figure 3：The notation of "wild type" and "WT" is mixed. It should be unified.

We have unified the use of WT in this figure.

Reviewer #3: Billmyre et al. attempt to globally identify genes ’contributing to sex’ in the fission yeast S. pombe. To this end, they use a Hermes transposon mutagenesis procedure coupled with next-generation sequencing developed by the Levin’s lab. Transposition is induced in a homothallic strain capable of self-mating. The mutagenized population is subjected to media changes to shift from vegetative growth to mating/sporulation and back to vegetative growth. Genes ‘contributing to sex’ are identified as non-essential genes that lack transposon integrations at the end of the regimen. 532 genes are identified in that way, of which 276 have prior ‘sexual fitness‘ annotations according to the authors tally. To get a feeling for how newly identified factors might function, the authors select four of them for more in-depth characterization: ifs1(SPAC3G6.03c), plb1 (SPAC1A6.04c), alg9 (SPAC1834.05), and sdg1 (SPAC12B10.02c).

I enjoyed many aspects of the manuscript. It addresses a biologically important question. The experiments were carefully conducted, varied approaches were used and the manuscript is attractively written.

Drawbacks are that evidence is lacking to support that the newly identified genes participate in ‘sex’ (comprising mating-type switching, pheromone sensing, cell fusion, nuclear fusion, meiosis, sporulation, and spore germination) rather than growth. This reduces the general interest of the study as only previously identified genes can be trusted hits. It also limits the value of the gene list as resource to a smaller community. A timeline indicating at which point each gene might be required would have helped, but such a timeline is missing. While well conducted, the detailed analysis of four candidate genes does little to counterbalance these issues.

We disagree with the reviewer that a clear line can be drawn between sexual reproduction and growth. Growth is required for mating-type switching, for extension of shmoos for mating, and for germination, all components of sexual reproduction. Our assay measures the relative fitness of mutants challenged to complete the whole process of sexual reproduction. Disrupting growth does decrease sexual fitness.

We do, however, understand the review’s point of view. For following up on our hits, we appreciate that many scientists will be more interested in genes specifically required for growth-independent reasons. For that reason, we have added in additional columns to Table S1 that capture two vegetative growth periods in our assay: from the original TN-seq library to our plated library and from our post germination short outgrowth to long outgrowth condition. We applied the same statistical approach that we used for our sexual reproduction assay to vegetative growth. We identified only 21 genes that were statistically “slow growing” (p < 0.05 after multiple test correction and negative fold change) before the sexual reproduction portion of the assay, 11 of which had previous annotations in sexual reproduction, and 46 that were “slow growing” after spore germination, 19 of which had previous annotations in sexual reproduction. Thus, instituting filtering resulted in loss of genes we knew were involved in sexual reproduction. Importantly, only 3 genes were slow at both time stages (*pvg1*, *meu10*, and *put2*) and two of those are annotated as sexual reproduction genes (*meu10* and *put2*).

We have modified Table S1 to include these data and have edited the results section of the text.

Could the study function as a useful resource? The approach is to some extent validated by the fact that many genes previously linked to mating/meiosis/spore formation and germination were identified. However, many previously identified genes are also missing, the concordance with previous studies is actually rather low.

Our approach identified new genes important for sexual reproduction, missed by previous analyses, which demonstrates the value of the contribution. We were not surprised at the low concordance of our work and the previous genome-wide screens. As discussed in the text, our study uses a different approach than other studies and required genes to cause a significant fitness defect to be hits. Notably, the previous studies also have low concordance with each other, but are still valuable contributions.

We acknowledge our assay also missed other genes known to contribute to reproduction. Some of these genes were likely missed because our assay focused on fitness and many characterized genes have relatively modest effects on fitness. In our discussion, we mention that one of the screens (and one of the primary sources of sexual reproduction annotations in *S. pombe*) set the threshold for “mutant” phenotype as <90% four spore tetrads. Spore viability remains very high in a population with such a mild phenotype and many of the more subtle mutants with this annotation would thus be invisible to our assay.

Many other genes known to contribute to sexual reproduction in *S. pombe* were first identified in other species, like *S. cerevisiae*, where their phenotypes are much stronger. For example, *hop1* contributes to meiosis in *S. pombe*, but a *hop1* deletion has only a mild effect on spore viability (89% of the wild-type fertility, PMID 20123974) and was not a significant hit in our assay. Other genes that contribute to reproduction may have been missed due to low insertion frequencies in our starting libraries. We extended the discussion of these possibilities in the text.

More worrisome, many of the novel hits are likely false positives. This is because the experimental design does not effectively distinguish genes required for sex from genes required for vegetative growth under the varied conditions used which include a staggering 9 days on MEA. Thus, genes are listed here as ‘important for sex’ while they are, most likely, just important for growth. The authors acknowledge 38 hits ‘putatively’ involved in metabolism (line 270 and Figure S4) but metabolic and biosynthetic pathways are much more prevalent, this can be seen in Table S1. Indeed, the adenine, arginine, asparagine, cysteine, histidine, isoleucine, lysine, proline, methionine, serine, and branched amino acids pathways are all represented. So are lipid metabolism, carbohydrate metabolism, and energy metabolism. But genes required for the biosynthesis of macromolecules are also abundant with numerous ribosomal proteins, ribosome biogenesis factors, translation factors, tRNA modifying enzymes, protein glycosylases and so forth. Altogether, these make up a large portion of the newly identified factors and include many of the factors conserved with vertebrates. Granted, some (and perhaps many) of these and other newly identified factors might be of particular importance for sexual reproduction (as plb1 or alg9 studied here, or gas4 for example which should probably be in the list of known factors), but this will require hard work to determine, one factor at a time, as no attempt was made here to experimentally refine the list in a global manner.

Thank you, we have adjusted the text and tables to highlight additional metabolic genes identified as hits. We specifically highlighted the categories suggested by the reviewer above using a representative GO term and all of its descendant terms (generation of precursor metabolites and energy (GO:0006091), lipid metabolic process (GO:0006629), carbohydrate metabolic process (GO:0005975), ribosome biogenesis (GO:0042254), translation (GO:0006412), tRNA metabolic process (GO:0006399), and protein modification process (GO:0036211) as well as deletions annotated with auxotrophy phenotypes (FYPO:0000128)). These annotations are now listed in Table S1.

65 of the 296 genes with existing sexual reproduction annotations (as defined in an answer below) also had “metabolic” annotations, meaning that this combination is not rare even in existing data. Further, 142 genes lacked existing annotations in any of these “metabolic” categories or in sexual reproduction. Of these, 98 were conserved in vertebrates.

Only 94 (17.7%) of our hits had one of the above “metabolic” annotations but lacked an existing sexual reproduction annotation, suggesting that even if these are false positives, they represent a limited portion of our hits. However, we disagree that these remaining hits with metabolism relevant annotations, but no prior sexual reproduction phenotype represent ‘false positives.’ See our comment above regarding distinguishing growth from sexual reproduction. We note that “important for growth” and “important for sex” are not mutually exclusive. Both *plb1* and *alg9* had existing “metabolic” annotations, but our experiment revealed novel sexual reproduction phenotypes for mutants of each gene.

We also note that amongst the ‘worrisome’ hits described above, the reviewer listed ribosomal proteins (RPs). We are actually investigating those hits as a follow up to this study because we noticed several cases where one ribosomal gene was a hit, but its paralog was not. The project is ongoing, but we have already confirmed one instance where one RP contributes strongly to sexual reproduction, but the paralog does not, despite both mutants having the same effect on growth rate. Nor is this limited to our data- two ribosomal subunits that were hits in our screen are already annotated as having defects in meiotic chromosome segregation (*rpl26*, *rps1702*) or mating (*rps101* and *rps1801*). The paralogs of these genes (*rps1701*, *rps102*, and *rps1802*) lack these sexual reproduction phenotype annotations.

Likewise, this is not limited to ribosomal proteins. Among other cases, *SPAC27E2.01* and *hmt2* are required for production of arginine and cysteine respectively, but mutants of either are also annotated as defective in sporulation or meiotic chromosome segregation, respectively. This highlights the value of agnostic genome-wide approaches and argues against over-filtering data to rule out genes predetermined to be ‘false positives.’

Also, we note that the 9 days on MEA was a purposeful part of our experimental design. This extended time allowed us to limit the selection against mutants that were slow to divide on MEA or were slow to complete sexual reproduction. The wild-type cells do not continue to expand over the course of 9 days. Instead, they divide to form a microcolony and complete sexual reproduction to form dormant spores within a couple of days. The extended time allowed slower mutants an opportunity to form spores at a slower pace without local competition from wild-type cells. This reduces the impact of the slow growth phenotypes the reviewer is concerned about on our assay.

We agree that follow-up work is required to understand how the genes we identified contribute to fitness in our assay, but this is an argument that can be applied to almost any genome-wide approach. We also agree that more experiments will help further narrow the point at which each of these factors may act, as described in the discussion, but believe this is outside the scope of the current work. We know that the presented work has made a valuable contribution to research in our group and feel it could help guide that of others.

Controls could have been instituted to distinguish ‘genes important for sex’ from ‘genes important for growth’ under the experimental media shifts, the issue cannot be addressed by a posteriori filtering. For instance, a mat1-deleted strain incapable of sex could have been processed in parallel with the h90 wild type, just skipping spore selection by glusulase treatment, to compare the list of genes devoid of transposon integrations in the two strains.

We appreciate this suggestion, and this experiment could prove useful in separating hits that are required for mating from those that are required post-mating. However, we disagree that this is an essential control for our experiments. One could imagine numerous potential secondary screens, including others detailed in the discussion, but we think the data presented provides a global view of the impact of the vast majority of non-essential genes to sexual reproduction.

Specific points:
How were the four genes ifs1(SPAC3G6.03c), plb1 (SPAC1A6.04c), alg9 (SPAC1834.05), and sdg1 (SPAC12B10.02c) selected for analysis? Were more genes examined, how were they selected and what was the outcome?

The three genes tested for sexual reproduction phenotypes (*ifs1*, *plb1*, and *alg9*) were selected based on four criteria:

1) They were statistically significant hits in our assay

2) They had very strong negative fold changes (ie. inserts went away after sex)

3) They were deleted in the original deletion collection, enabling us to simply PCR amplify deletion cassettes to confirm our phenotypes in a clean background

4) They lacked annotated negative sexual reproduction phenotypes in Pombase

We specifically sought out genes to test that were assayed in previous screens and did not appear to be involved in sexual reproduction in previous screens (ie. biasing ourselves towards those most likely to be incorrect in our own screen). We still found phenotypes for all three of these mutants that explained their behavior in our assay, demonstrating its ability to detect phenotypes that were missed by the field.

*sdg1* was one of two putatively essential genes we selected that were missing from the deletion collection and had strong phenotypes in our assay. We were able to delete only *sdg1*, as described in the text. We suspect the other was truly essential.

Aside from our borderline hit *atg11*, there were no other hits from our assay tested for this manuscript (ie., we did not test 20 hits, fail to find a phenotype for most, and then not report them).

Figure S4 presents a Venn Diagram with 38 ‘annotated metabolic genes’ and 211 genes ‘without relevant annotations’. Could the authors please clarify the definition of ‘relevant’ by providing gene lists (including protein functions and GO terms) for each category in this Venn diagram. This information can be retrieved from PomBase and could perhaps simply be added to Table S1.

These annotations were originally based on manual annotation using the PomBase “single locus phenotype” annotations for deletions. We have gone back through the gene lists to add the requested annotations to Table S1 as requested. Rather than manual annotation, we have redone the annotation systematically using scripts after selecting a uniform set of relevant phenotype and GO annotations as described below. This resulted in a revised Figure S4 and Table S1.

For phenotype annotations, we manually selected sexual reproduction relevant phenotypes from the set of FYPO annotations, resulting in a set of 165 unique phenotype annotations, now available as Supplemental File 1. We then searched this list against the phenotype annotations from Pombase (downloaded on 8/7/22) for our set of hits, including only annotated phenotypes for deletion mutants to avoid the possibility of point mutants not representing loss of function phenotypes. For GO term analysis, we searched for existing annotations for three parent GO terms (Mating-type Determination, Meiotic cell cycle, Sexual Reproduction) and all of their descendant GO terms. This covered a total of 688 unique GO terms, including the three parent terms.

In combination with the metabolic annotation described above, this new analysis resulted in 136 hits without relevant annotations (sexual reproduction, metabolic/biosynthetic genes, or essential genes) rather than the 211 previously reported. There are a few discrepancies between the annotation raw data files provided by Pombase and the information available for those genes on the Pombase website, meaning that our systematic approach no longer reports some genes as sexual reproduction genes that are currently listed as such on the website (*npp106* and *pom152*). As the reviewer pointed out above, we did not originally annotate *gas4* as a sexual reproduction gene and should have. This is because it lacked a “single locus phenotype” annotation relevant to its described role in forming spore walls, despite this role clearly being represented in the literature. Alternately, other genes, such as *swi1,* were missed because none of the alleles are annotated as deletion alleles, despite *swi1* having a classically defined role in mating type switching. Fortunately, this analysis is complemented by the added GO term analysis which captures the *gas4* and *swi1* sexual reproduction roles. This problem also applied to the new automated assessment of auxotrophy, as many classic auxotrophic mutants were not assessed using deletion alleles and thus were not scored as such here.

We have added text to the methods and edited the results accordingly.

Minor points:
Overreaching statement: Line 521 ‘the quorum sensing pathway we have identified here’ is overreaching as no quorum sensing pathway has been identified.

We agree with the reviewer and have changed this text to “quorum-regulated phenotype.”

The author should use ‘medium’ rather than ‘media’ when referring to a single medium.

We agree with the reviewer and have made this change.
